# Gammaherpesvirus-Driven Plasma Cell Differentiation Regulates Virus Reactivation from Latently Infected B Lymphocytes

**DOI:** 10.1371/journal.ppat.1000677

**Published:** 2009-11-26

**Authors:** Xiaozhen Liang, Christopher M. Collins, Justin B. Mendel, Neal N. Iwakoshi, Samuel H. Speck

**Affiliations:** 1 Department of Microbiology & Immunology, Emory University School of Medicine, Atlanta, Georgia, United States of America; 2 Emory Vaccine Center, Emory University School of Medicine, Atlanta, Georgia, United States of America; 3 Department of Surgery, Emory University School of Medicine, Atlanta, Georgia, United States of America; University of North Carolina Chapel Hill, United States of America

## Abstract

Gammaherpesviruses chronically infect their host and are tightly associated with the development of lymphoproliferative diseases and lymphomas, as well as several other types of cancer. Mechanisms involved in maintaining chronic gammaherpesvirus infections are poorly understood and, in particular, little is known about the mechanisms involved in controlling gammaherpesvirus reactivation from latently infected B cells in vivo. Recent evidence has linked plasma cell differentiation with reactivation of the human gammaherpesviruses EBV and KSHV through induction of the immediate-early viral transcriptional activators by the plasma cell-specific transcription factor XBP-1s. We now extend those findings to document a role for a gammaherpesvirus gene product in regulating plasma cell differentiation and thus virus reactivation. We have previously shown that the murine gammaherpesvirus 68 (MHV68) gene product M2 is dispensable for virus replication in permissive cells, but plays a critical role in virus reactivation from latently infected B cells. Here we show that in mice infected with wild type MHV68, virus infected plasma cells (ca. 8% of virus infected splenocytes at the peak of viral latency) account for the majority of reactivation observed upon explant of splenocytes. In contrast, there is an absence of virus infected plasma cells at the peak of latency in mice infected with a M2 null MHV68. Furthermore, we show that the M2 protein can drive plasma cell differentiation in a B lymphoma cell line in the absence of any other MHV68 gene products. Thus, the role of M2 in MHV68 reactivation can be attributed to its ability to manipulate plasma cell differentiation, providing a novel viral strategy to regulate gammaherpesvirus reactivation from latently infected B cells. We postulate that M2 represents a new class of herpesvirus gene products (reactivation conditioners) that do not directly participate in virus replication, but rather facilitate virus reactivation by manipulating the cellular milieu to provide a reactivation competent environment.

## Author Summary

Gammaherpesviruses are associated with the development of lymphomas, particularly in immunosuppressed individuals, as well as several other types of cancers. Like all herpesviruses, once a host is infected these viruses cannot be cleared and, as such, infected individuals harbor these viruses for life. One of the important strategies utilized by herpesviruses to chronically infect their host is their ability to establish a largely quiescent form of infection referred to as latency, in which no progeny virus is produced. Importantly, all herpesviruses have the capacity to emerge from latency and replicate, a process referred to as reactivation. Gammaherpesviruses largely persist in a population of white blood cells called B lymphocytes which, upon differentiation into plasma cells, produce antibodies in response to infection. Notably, it has been recently shown for the human gammaherpesviruses, Epstein-Barr virus and Kaposi's sarcoma-associated herpesvirus, that virus reactivation from latently infected B lymphocytes involves differentiation of the infected B lymphocytes to plasma cells. Here, using a small animal model of gammaherpesvirus infection, we show that plasma cell differentiation is also associated with reactivation of murine gammaherpesvirus 68. Furthermore, we show that this requires a protein encoded by the virus which is able to drive plasma cell differentiation. Thus, our studies not only confirm the importance of plasma cell differentiation in gammaherpesvirus reactivation from B lymphocytes, but also provide evidence that this process is controlled by a viral protein.

## Introduction

Plasma cells, which are the cellular factories that produce secreted antibody, play a critical role in mounting an effective immune response to many pathogens. Early plasma cell responses to foreign antigens can be divided into two phases: (i) differentiation of short-lived plasma cells arising from naïve marginal-zone and mature follicular B cells, which secrete low affinity antibodies that have not undergone somatic mutation and are thought to provide an initial rapid response to the invading pathogen; and (ii) differentiation of follicular B cells upon encountering antigen and receiving T cell help, leading to the formation of germinal centers, several rounds of B cell proliferation, affinity maturation, class-switching, and ultimately the development of memory B cells and plasma cells that serve to sustain humoral immune responses [1]. Although the signal(s) that initiate plasma cell differentiation remain controversial, recent progress has identified several critical transcriptional regulators of plasma cell differentiation - including B lymphocyte induced maturation protein 1 (Blimp-1), interferon regulatory factor-4 (IRF-4) and XBP-1s [2],[3],[4],[5],[6].

Crawford and Ando [7] provided early evidence that Epstein-Barr virus (EBV) replication-associated antigens were present in Burkitt's lymphoma cells that exhibited a plasma cell phenotype – providing the first evidence that plasma cell differentiation is associated with virus reactivation from latency. More recently this observation has been extended to show that plasma cell differentiation is associated with reactivation of both EBV and Kaposi's sarcoma-associated herpesvirus (KSHV). Induction of EBV and KSHV replication in latently infected B cells appears to be driven by the plasma cell-specific transcription factor XBP-1s, which activates transcription of the viral immediate-early genes encoding the critical transcriptional activators that trigger the EBV and KSHV replication cascades (BZLF1 and BRLF1/gene 50 in the EBV genome and gene 50 in the KSHV genome) [8],[9],[10],[11],[12]. Whether plasma cell differentiation leading to virus reactivation is a common strategy utilized by B cell-tropic gammaherpesviruses remains to be determined.

Murine gammaherpesvirus 68 (MHV68) infection of mice provides a tractable small animal model to investigate basic issues of gammaherpesvirus pathogenesis. Previous characterizations of MHV68 latency in mice have shown that B cells, as well as some populations of macrophages and dendritic cells, harbor latent virus - with B cells appearing to represent the major long term latency reservoir [13],[14],[15],[16],[17]. Analyses of virus latency in the spleen have shown that during the establishment of latency MHV68 is found in naive, germinal center and memory B cells [14],[18]. However, latency in naive and germinal center B cells wanes with time and at late times post-infection MHV68, like EBV, is predominantly found in memory B cells [14],[18]. Recently, we have also identified MHV68 in plasma cells at the peak of virus latency in the spleen [19]. The latter observation raises the possibility that, like EBV and KSHV, plasma cell differentiation may be associated with MHV68 reactivation.

Here we demonstrate that plasma cell differentiation is linked to MHV68 reactivation from latency. In addition, we provide evidence that a MHV68 encoded gene product, M2, plays a seminal role in virus gaining access to plasma cells. We have previously shown that the MHV68 M2 protein, which is expressed in a subset of infected B cells at the peak of viral latency [20], plays critical roles in both the establishment of latency as well as virus reactivation from latently infected B cells - phenotypes that are influenced by both route and dose of virus inoculation [18],[21]. Additionally, efficient transition of latently-infected B cells from the germinal center reaction to the memory B cell reservoir appears to be stalled in the absence of M2, suggesting M2 may manipulate B cell signaling or differentiation to facilitate establishment of long-term latency in the memory B cell pool [18],[22]. Importantly, M2 is dispensable for virus replication in permissive fibroblasts in vitro or during acute virus replication in the lungs following intranasal inoculation [21]. Thus, M2 appears to have a specialized role during establishment of latency and reactivation. M2 contains several SH3 domain docking sites, along with 2 functionally important tyrosine residues that are targets for phosphorylation, and is thought to function as a molecular scaffold that modulates B cell signaling pathways [23],[24]. Notably, M2 has been shown to target phosphorylation of the guanosine nucleotide exchange factors Vav1 and Vav2, and more recently has been shown form a trimolecular complex with Vav1 and the Src family tyrosine kinase Fyn [24] - although the functional consequences of these interactions remain unclear. Here we focus on the role of M2 in virus reactivation from B cells.

## Results

### M2 is required for efficient virus reactivation from latently infected B cell lines in vitro

**Figure 1:**
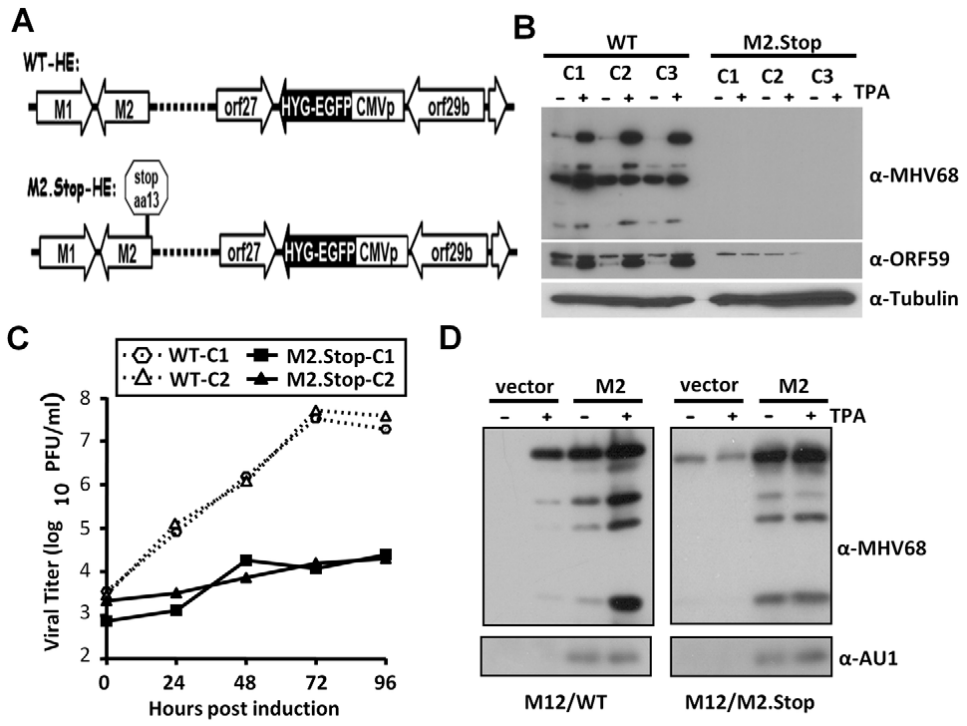
Requirement of MHV68 M2 protein for efficient reactivation from B cell latency. (A) Schematic illustration MHV68 recombinant viruses harboring a hygromycin phosphotransferase gene fused to EGFP (Hygro-EGFP) in the neutral locus of either wild type (WT-HE) or an M2-null mutant virus (M2.Stop-HE). (B) Latently infected WT-HE and M2.Stop-HE M12 cells were either mock treated or stimulated with TPA and expression of viral replication-associated antigen detected with either an anti-MHV68 serum generated in infected rabbits or antibodies raised against a peptide from the replication-associated ORF59 encoded viral antigen. Antibodies against tubulin were used to control for amount of cell lysate loaded. (C) Virus production from WT-HE and M2.stop-HE M12 cells as a function of time post-TPA stimulation. Virus titers were determined by plaque assay on NIH 3T3 fibroblasts as previously described [38]. (D) WT or M2.Stop-HE M12 cells were either mock treated or stimulated with TPA at 24 hr post-transfection with an M2 expression plasmid (pEF/M2-AU1) or the parental empty expression vector (pEF). Cell lysates harvested at 24 hr post-TPA induction were analyzed by immunoblotting using a rabbit anti-MHV68 antiserum generated from MHV68 infected rabbits [38].

To begin characterizing the role that M2 plays in virus reactivation from latently infected B cells, we initially determined whether a requirement for M2 in MHV68 reactivation from latently infected B cells could be recapitulated in a tissue culture B cell latency model. Utilizing the murine M12 B lymphoma cell line, we generated a number of clonal cell lines harboring either a recombinant wild type MHV68 containing a hygromycin-GFP fusion protein expression cassette, or a M2-null MHV68 mutant (M2.Stop) on the same genetic background (Fig. 1A). Stable cell lines were generated using hygromycin selection, and then analyzed for virus reactivation following stimulation with the phorbol ester TPA. Notably, TPA treatment induced significant virus reactivation in all the M12 clones infected with wild type MHV68 - as shown by both immunoblot analyses of expression of MHV68 replication-associated antigens (Fig. 1B) and increased titers of infectious virus in the tissue culture supernatants (Fig. 1C). In contrast, TPA treatment of M12 cell lines infected with the M2.Stop virus resulted in little increase in virus replication (Fig. 1B and 1C). Similarly, treatment with either 5-azacytidine or trichostatin A was able to induce reactivation of the wild type MHV68 M12 cell lines, but not the M2.Stop infected clones (data not shown). Importantly, ectopic expression of M2 rescued virus reactivation from the M2.Stop infected M12 cell lines (Fig. 1D) - indeed, expression of M2 alone in the absence of TPA induction resulted in a significant increase in the expression of replication-associated viral antigens from both wild type and M2.Stop infected M12 cell lines. The ability of ectopic M2 expression to drive MHV68 reactivation was not limited to the latently infected M12 cell lines, but independently observed with latently infected murine A20 B lymphoma cell lines (data not shown). The latter results suggest a direct link between M2 expression and MHV68 reactivation from latently infected B cells.

Although M2 is completely dispensable for MHV68 replication in permissive cell lines [18],[21], we next assessed whether it might play a B cell-specific role in activating transcription from the immediate-early gene 50 promoter which encodes the essential lytic switch protein RTA. To assess whether M2 could directly activate the immediate-early gene 50, we used reporter constructs in which the proximal gene 50 promoter was cloned upstream of a firefly luciferase reporter gene. We have previously shown that the proximal gene 50 promoter is required for reactivation of MHV68 from latently infected B cells [25]. Notably, M2 expression (in the absence of other viral proteins) could only weakly upregulated (<3-fold) gene 50 promoter activity (data not shown), indicating that the role of M2 in virus reactivation from the latently infected M12 B cell lines is unlikely to be via direct targeting/regulation of gene 50 transcription. As such, we turned our attention to further investigating the impact of loss of M2 expression on virus infection in vivo.

### Wild type and M2 null MHV68 exhibit a similar distribution in marginal zone, follicular and newly formed B cell populations in the spleen at the peak of virus latency

**Figure 2:**
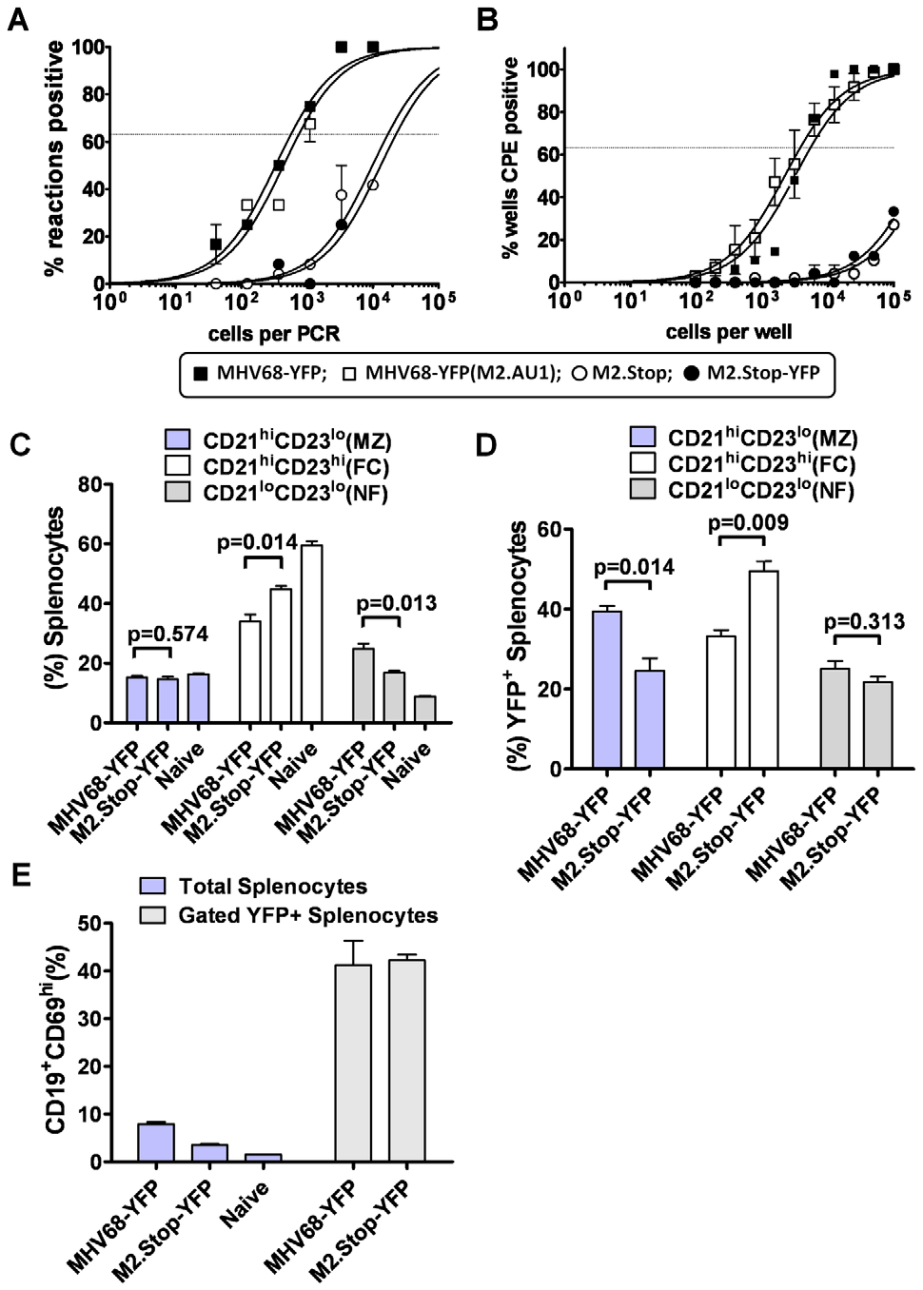
Distribution of M2 null virus in newly formed, marginal zone and follicular B cells in the spleen at the peak of latency is similar to wild type MHV68. (A and B) Insertion of a YFP expression cassette into the viral genome, or addition of an AU1 epitope tag on the C-terminus of M2, did not alter the phenotype of the M2 null virus. Latency and reactivation phenotypes of several YFP expressing MHV68 recombinant viruses were assessed using standard limiting dilution analyses to compare the eYFP expressing M2.Stop recombinant virus to the well characterized M2.Stop mutant, as well as different wt YFP expressing viruses - one of which contains an AU1 epitope tag on the C-terminus of M2. All data were pooled from two independent virus isolate, five mice infected with 100 pfu of the indicated virus via intranasal inoculation. (A) Limiting dilution PCR analyses of the frequency of splenocytes harboring viral genome following infection with the indicated recombinant viruses. (B) Limiting dilution analyses of MHV68 reactivation following explant of splenocytes harvested at day 16 post-infection. (C) Similar levels of marginal zone (MZ), follicular (FC) and newly formed (NF) B cells in spleens infected with wt MHV68-YFP or M2 null viruses (M2.stop-YFP). (D) Percentage of MZ, FC and NF B cells in virus infected (YFP+ cells) splenocytes with wt MHV68-YFP or M2 null viruses. (E) Percentage of activated B cells present in wt MHV68-YFP or M2 null virus infected spleen cells at day 16 post-infection. The p values indicated in the figure were determined by two-tailed, unpaired *t* test with a 95% confidence level.

To track MHV68 latently infected B cell populations in vivo, we generated a recombinant MHV68 harboring an enhanced yellow fluorescent protein (eYFP) transgene under the control of the HCMV immediate-early promoter and enhancer (MHV68-YFP) [19]. We have extensively characterized the MHV68-YFP recombinant virus and have shown that it behaves like wild type virus and is able to efficiently mark latently infected cells at the peak of viral latency (days 16–18 post-infection) [19]. For our studies on M2 function we generated an M2 null MHV68 harboring the eYFP expression cassette (M2.Stop-YFP), which is described below, as well generating and characterizing a recombinant MHV68 with an AU1 epitope tag fused to the C-terminus of M2 [MHV68-YFP(M2.AU1)]. To ensure that insertion of the eYFP expression cassette did not adversely impact the M2 null virus phenotype, we compared infection of mice with either the previously characterized M2.Stop mutant virus [18], the M2.Stop-YFP virus, or MHV68-YFP virus, followed by analysis of establishment latency and reactivation at day 16 post-infection. In addition, since the analyses of M2 function discussed above used an M2 expression vector in which an AU1 epitope tag was inserted at the C-terminus of M2, we also generated and characterized a recombinant MHV68 in harboring the AU1 epitope tagged M2 [MHV68-YFP(M2.AU1)]. These analyses demonstrated that the phenotype of the eYFP expressing M2 null virus was indistinguishable from the well characterized M2.Stop mutant virus (Fig. 2A and 2B). In addition, the insertion of an AU1 epitope tag did not appear to have any impact on the phenotype of the MHV68-YFP virus (Fig. 2A and 2B).

We next sought to characterize and compare B cell populations harboring wild type or M2 null MHV68 (M2.Stop) in infected mice. The infected spleens were harvested at day 16 post-infection and the presence of virus in distinct B cell populations was assessed by flow cytometry. Analyses of total splenoctyes revealed equivalent levels of marginal zone B cells (CD21^hi^CD23^lo^) in infected and naïve mice, but slightly lower levels of follicular B cells (CD21^hi^CD23^hi^) coupled with a slight increase in newly formed B cells (CD21^lo^CD23^lo^) in mice infected with either wild type or M2 null MHV68 compared to naïve mice (Fig. 2C). Analysis of the distribution of virus in these B cell populations revealed that M2 null virus infection, like wild type MHV68, was present in each of these B cell populations (Fig. 2D). However, the M2 null virus was diminished in marginal zone B cells and elevated in follicular B cells compared to wild type MHV68 (Fig. 2D). As we have previously noted [19], the pattern of CD21 and CD23 surface expression on MHV68 infected cells is somewhat distinct from that observed in naïve mice. Indeed, it has been shown that both CD21 and CD23 expression can be modulated by EBV, KSHV and MHV68 [26],[27] (C.M. Collins and S.H. Speck., unpublished data). Thus, some caution must be taken in interpreting the distribution of wild type and M2 null MHV68 in these splenic B cell populations. Finally, although we observed decreased levels of activated B cells (CD19^+^CD69^hi^) in the spleens of mice infected with the M2 null virus compared to wild type MHV68-YFP infected mice (which correlates with a less robust establishment of splenic latency in M2.Stop infected mice), a similar percentage (ca. 40%) of virus infected B cells (YFP+) exhibited an activated phenotype in M2.Stop-YFP and wild type MHV68-YFP infected mice (Fig. 2E).

### M2 is not required for entry of MHV68 latently infected B cells into germinal centers, but the absence of M2 leads to alterations in immunoglobulin isotype class switching

**Figure 3:**
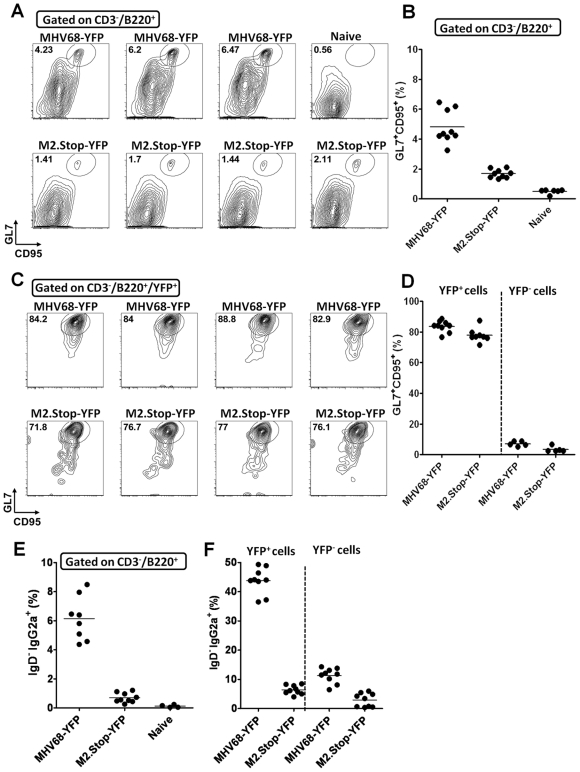
M2 null virus infected B cells form germinal centers, but exhibit a defect in immunoglobulin isotype class switching. Mice were infected with a 100 pfu of either MHV68-YFP or M2.Stop-YFP viruses via intranasal inoculation. (A) Total germinal center B cells (CD3^−^/B220^+^/GL7^hi^/CD95^hi^) present in the spleen are reduced in M2.stop-YFP infected mice compared to MHV68-YFP infected mice at day 16. Representative flow cytometry plots are shown for 3–4 individual mice. (B) Scatter plot depicting the percentage of total B220+ splenocytes that are germinal center B cells (GL7+/CD95+) in naïve mice, MHV68-YFP or M2stop-YFP infected spleens at day 16 post-infection. (C) The distribution of wild type and M2 null virus infected B cells (YFP^+^ cells) in the spleens of infected mice. (D) Percentage of wild type and M2 null virus infected (YFP+) B cells vs uninfected B cells (YFP-) in the spleen that exhibited a germinal center phenotype. (E) Percentage of splenic B cells that were IgG2a+ in wild type vs M2 null virus infected mice at day 16 post-infection. (F) M2.stop-YFP^+^ spleen cells showed an impaired ability to class-switch to IgG2a.

Comparing establishment of B cell latency in the spleen using wild type MHV68-YFP and M2.Stop-YFP recombinant viruses, we observed a smaller germinal center response in mice infected with the M2.Stop-YFP virus at day 16 post-infection (Fig. 3A and 3B). However, even though there was a diminished germinal center response (coupled with a lower frequency of M2.Stop-YFP infected B cells compared to MHV68-YFP infected mice), when we examined the distribution of virus infected cells we observed that a very similar percentage of wild type and M2.Stop virus infected B cells exhibited a germinal center phenotype (GL7^+^/CD95^+^) (Fig. 3C and 3D). This substantiates earlier analyses demonstrating the ability of M2 null viruses to form and expand within germinal centers [18],[22].

Examination of immunoglobulin isotype class switching in the spleens of MHV68-YFP and M2.Stop-YFP infected mice revealed a significant difference in the presence of B cells that had switched to IgG2a, the predominant IgG subtype observed following MHV68 infection (unpublished data) as well as many other viral infections [28]. While approximately 6% of splenic B cells were IgD−/IgG2a+ following MHV68-YFP infection, <1% had switched to IgG2a following infection with the M2.Stop-YFP virus (Fig. 3E). Furthermore, approximately 50% of wild type MHV68-YFP was found in IgG2a+ B cells while <10% of M2.Stop-YFP virus was in IgG2a+ B cells (Fig. 3F). These results are consistent with our previous analyses indicating that the M2.Stop virus is impaired in gaining access to isotype switched B cells [18]. These results implicate a role for M2 in modulating immunoglobulin isotype class switching.

### M2 is required for differentiation of MHV68 infected B cells to plasma cells

**Figure 4:**
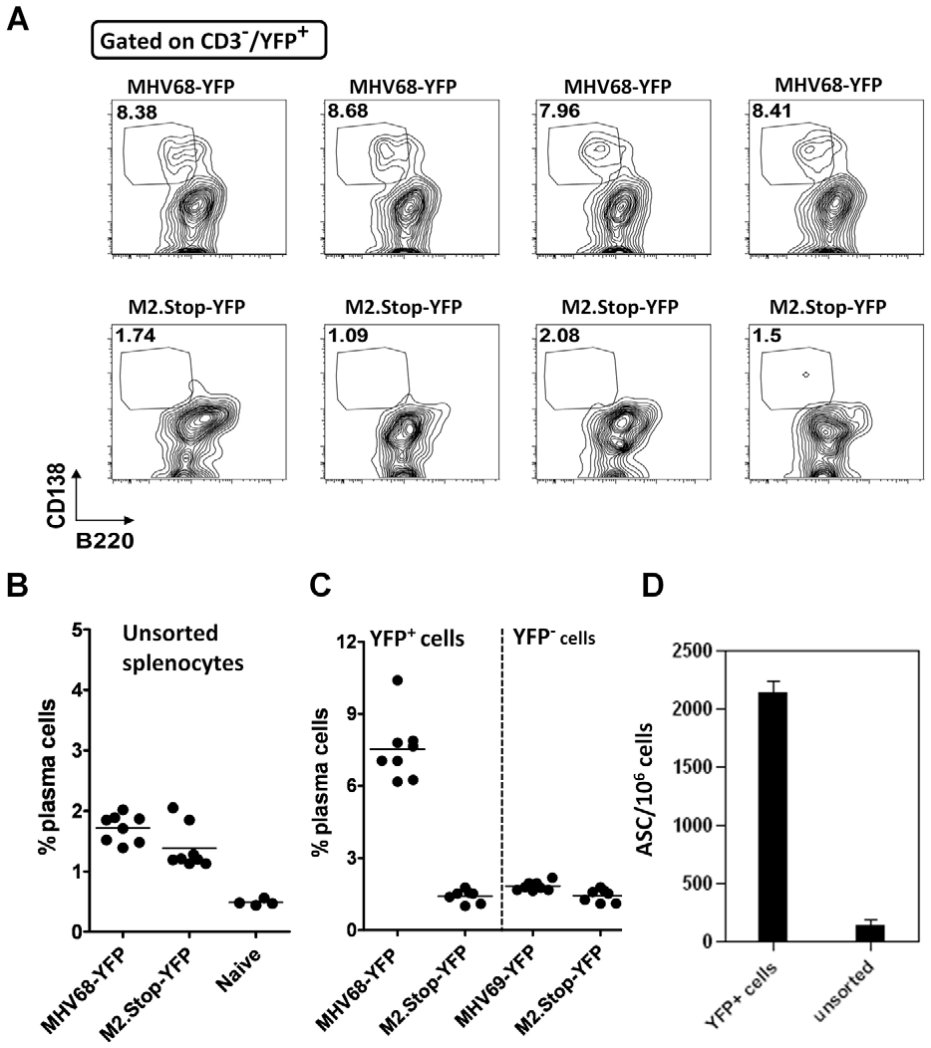
Access of MHV68 to the plasma cell reservoir during MHV68 chronic infection requires a functional M2 gene. Mice were infected with a 100 pfu of either MHV68-YFP or M2.Stop-YFP viruses via intranasal inoculation. (A) The absence of MHV68 infected plasma cells in mice infected with the M2.Stop-YFP virus. Flow cytometry analyses, gated on YFP+ cells, to determine the presence of virus infected plasma cells (CD138^+^/B220^−^) in the spleen at day 16 post-infection. Data shown were compiled from representative wild type (MHV68-YFP) and M2 null virus (M2.Stop-YFP) infected mice (4 mice per virus). (B) Similar levels of total plasma cells in mice infected with wild type vs. the M2 null MHV68. Compiled data showing the percentage of plasma cells present in wt MHV68 (MHV68-YFP) infected or M2 null virus (M2.stop-YFP) infected spleen cells at day 16 post-infection. (C) Percentage of plasma cells in virus infected (YFP+) and uninfected (YFP-) splenocytes harvested at day 16 post-infection. (D) ELISPOT analyses of antibody secreting cells (ASC) present in wt MHV68 (MHV68-YFP) infected splenocytes (YFP+ cells) or bulk splenocytes harvested at day 16 post-infection.

Based on the recently established link between plasma cell differentiation and gammaherpesvirus reactivation [8],[9],[10],[11],[12], we assessed the presence of MHV68 in plasma cells at the peak of splenic latency (days 16–18 post-infection). We analyzed individual mice infected with wild type MHV68-YFP or M2.Stop-YFP for the presence of virus infected plasma cells (YFP+/B220^lo/−^/CD138^+^). Consistently, in mice infected with MHV68-YFP, approximately 8% of virus infected B cells were plasma cells at day 16 post-infection (Fig. 4A and 4C). In stark contrast, ≤1.5% of M2.Stop-YFP virus infected B cells were plasma cells (Fig. 4A and 4C). Notably, analysis of bulk splenocytes revealed that the frequency of plasma cells in the spleens of wild type and M2 null virus infected mice were relatively comparable (approximately 1.5%), and slightly higher than the levels observed in naive mice (about 0.5%) (Fig. 4B). Finally, we used a functional assay to directly assess the presence of wild type MHV68 in plasma cells. YFP+ splenocytes were isolated by flow cytometry and analyzed by ELISPOT to determine the frequency of antibody secreting cells (ASC). As expected, the YFP+ cell population recovered from mice infected with the wild type MHV68-YFP virus was substantially enriched for plasma cells compared to unfractionated splenocytes (Fig. 2D). Because we have previously shown that dose of M2.Stop virus can impact the phenotype observed [18], we assessed whether increasing the dose of M2.Stop from 100 to 1,000 pfu would lead to detectable M2.Stop virus infection of plasma cells. Notably, even following intranasal inoculation with 1,000 pfu of M2.Stop-YFP we failed to observe any YFP+ plasma cells (data not shown). Thus, we conclude that a significant percentage of MHV68 infection at the peak of viral latency is present in plasma cells, and that this population is largely absent in mice infected with the M2 null virus (M2.Stop-YFP), indicating a critical role for M2 in plasma cell differentiation during MHV68 chronic infection.

### Plasma cells account for the majority of MHV68 reactivation from explanted splenocytes

**Figure 5:**
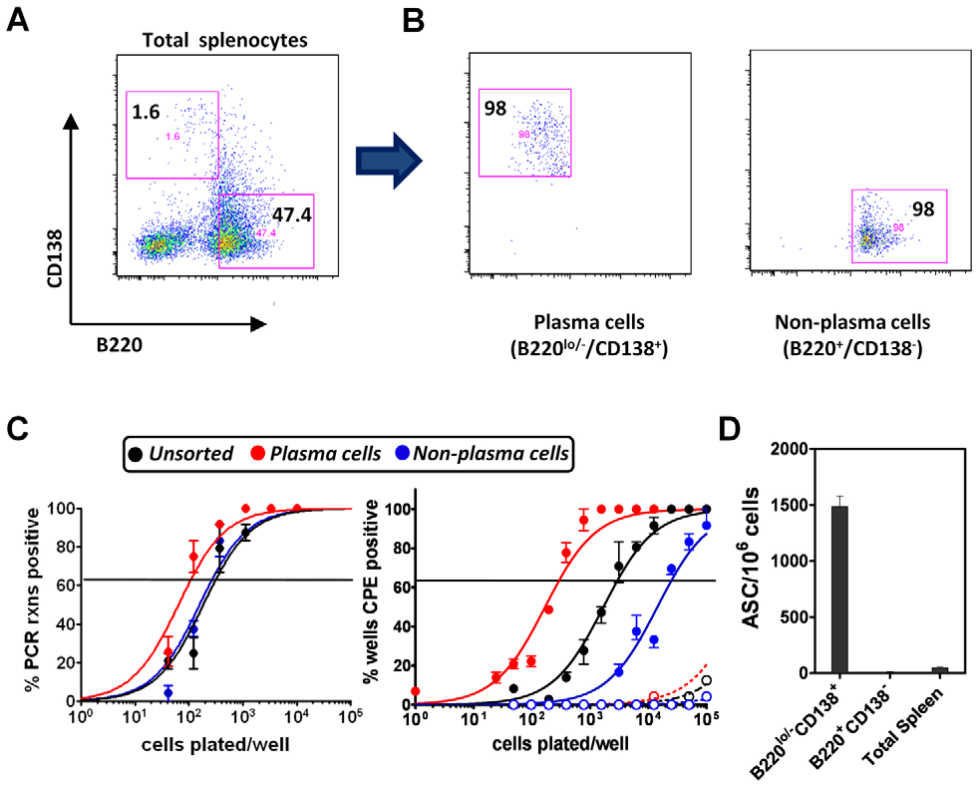
Plasma cells contribute significantly to spontaneous MHV68 reactivation following explant. (A and B) Purification of plasma cells and non-plasma cells by flow cytometry. Shown are representative flow plots unsorted (A) and post-sorting (B), as well as the gates used to isolate the plasma cells and non-plasma cell populations. Cells were sorted from MHV68-YFP infected spleens at day 16 post-infection. (C) Limiting dilution determinations of the frequency of splenocyte populations harboring the MHV68 genome (left panel) or spontaneously reactivating virus upon explant (right panel). Splenocytes were harvested from C57Bl/6 mice at day 16 post-infection. For the reactivation analyses, both intact cells (filled symbols) and mechanically disrupted cells (open symbols) were plated to distinguish the presence of pre-formed infectious virus from reactivating virus. (D) ELISPOT analyses of antibody secreting cells in the unsorted and purified plasma cell and non-plasma cell populations analyzed in (A).

We noted that the percentage of virus infected splenocytes that were plasma cells (see Fig. 4C) correlated closely with the percentage of infected splenocytes that spontaneously reactivate MHV68 upon explants [13]. To assess whether MHV68 reactivation is linked to plasma cell differentiation, we purified plasma cells from mice infected with wild type MHV68-YFP and simultaneously isolated plasma cell-depleted splenocytes (Fig. 5A and 5B). Subsequent ELISPOT analyses of the purified populations confirmed appropriate enrichment or depletion of plasma cells (Fig. 5D).

To determine the frequency of cells in each population harboring viral genome (a surrogate measure of the frequency of latently infected cells), the plasma cell enriched and depleted populations, along with unfractionated splenocytes, were analyzed for the presence of MHV68 infection using a limiting dilution nested PCR analysis [13] (Fig. 5C, left panel). This analysis revealed that there was a slightly higher frequency of viral genome positive cells in the plasma cell population (approximately 1 in 100 plasma cells) than in either total splenocytes or plasma cell depleted splenocytes (approximately 1 in 300 cells). Virus reactivation was examined using a limiting dilution analysis in which splenocyte populations were plated onto permissive monolayers of mouse embryo fibroblasts and virus reactivation scored 2 to 3 weeks post-plating by the appearance of viral cytopathic effect (cpe) [13]. Notably, this reactivation analysis revealed a profound difference between the plasma cell enriched and depleted populations (Fig. 5C, right panel). Approximately 50% of MHV68 infected plasma cells spontaneously reactivated virus in this assay (approximately 1 in 200 plasma cells reactivated virus compared to 1 in 100 plasma cells which harbor viral genome), while only approximately 1% of the virus infected non-plasma cell population reactivated virus (approximately 1 in 25,000 non-plasma cells reactivated virus compared to 1 in 300 in this population that harbor viral genome). As expected, approximately 10% of unfractionated splenocytes spontaneously reactivated virus (approximately 1 in 3,000 splenocytes reactivated virus compared to 1 in 300 harboring viral genome) (Fig. 5C, right panel). Thus, even though the frequency of infected cells is roughly the same in the plasma cell and non-plasma cell splenocyte populations, the frequency of plasma cells reactivating virus is ca. 100-fold higher than the non-plasma cell fraction. These results are consistent with recent studies characterizing EBV and KSHV reactivation that have linked plasma cell differentiation to virus reactivation [8],[9],[10],[11],[12]. In light of these results, the nearly complete absence of the M2 null mutant (M2.Stop-YFP virus) in splenic plasma cells correlates with the B cell reactivation defect observed with this mutant virus.

### M2 is sufficient to drive plasma cell differentiation of the BCL-1 B lymphoma cell line

**Figure 6:**
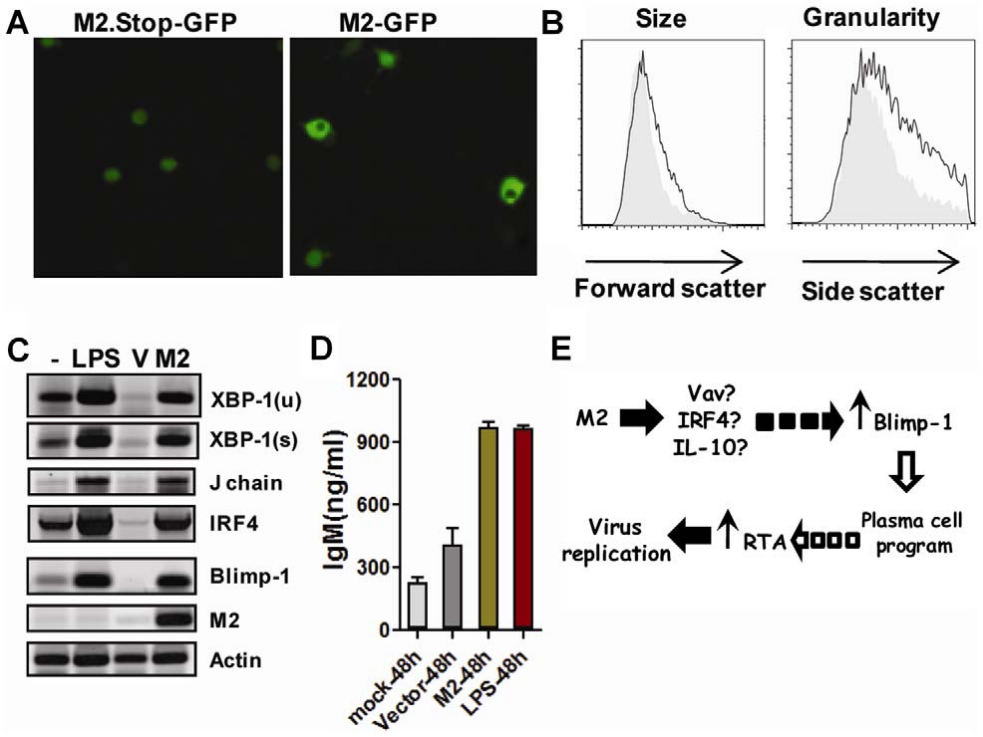
M2 can drive plasma cell differentiation in a B lymphoma cell line. (A) Appearance of plasmacyte morphology following transfection of the murine BCL-1 lymphoma cells with a M2 expression vector. GFP fluorescence in BCL-1 lymphoma cells transfected with either vector control (MSCV-M2.Stop-IRES-GFP) or an M2 expression vector (MSCV-M2-IRES-GFP). GFP expression, driven from an internal ribosome entry site introduced downstream of the M2 open reading frame, was examined at 24 hr post-transfection. (B) M2 expressing BCL-1 cells exhibited increased size and granularity compared to M2.Stop transfected cells, as determined by changes in forward and side light scatter. Filled gray histograms were gated on the GFP expressing cell population in BCL-1 cells transfected with the M2.Stop vector, while the black open histograms were gated on the GFP expressing cell population in BCL-1 cells transfected with the M2 expression vector. (C) RT-PCR analysis of transcripts associated with the plasma cell differentiation program. -, untreated BCL-1 cells; LPS, BCL-1 cells treated with 20 ug/ml LPS and RNA prepared at 24 hr post-treatment; V, BCL-1 cells transfected with a control vector (MSCV-M2.Stop-IRES-GFP); M2, BCL-1 cells transfected with an M2 expression vector (MSCV-M2-IRES-GFP). BCL-1 cells were transfected using a Nucleofector (Amaxa) and RNA prepared at 24 hours post-transfection. (D) Analysis of IgM levels in the tissue culture supernatant of mock treated vs LPS stimulated, or vector control (MSCV-M2.Stop-IRES-GFP) vs M2 expression vector (MSCV-M2-IRES-GFP) transfected BCL-1 cells. As indicated, IgM levels were determined at 48 hours post-treatment. (E) Model of M2 induction of plasma cell differentiation leading to virus reactivation from latency.

To determine whether M2 plays a direct role in driving plasma cell differentiation, we utilized the BCL-1 B lymphoma cell line which can be induced to differentiate into plasma cells by various stimuli [3],[29]. Transient transfection of murine stem cell virus (MSCV) vectors containing either M2 or a negative control (MSCV harboring the M2.Stop expression cassette) into the BCL-1 cell line resulted in an M2-dependent change in cell morphology (Fig. 6A). M2 expression (monitored by GFP expression) led to acquisition of a plasmacyte morphology (Fig. 6A), which could be detected by flow cytometry as an increase in both size and granularity of the cells (Fig. 6B).

We extended this analysis to examine changes in the expression of genes associated with the plasma cell differentiation program. Compared to M2.Stop transfected cells, there was a significant induction in the levels of transcripts encoding several plasma cell-associated factors (XBP-1s, Blimp-1, J chain and IRF-4); changes that were also observed upon LPS stimulation of the BCL-1 cell line (Fig. 6C). Notably, we repeatedly observed, following transfection with the M2.Stop vector, lower levels of these plasma cell-associated transcripts than was observed in untreated BCL-1 cells – suggesting that the transfection protocol leads to selective loss of those cells in the starting BCL-1 cell population that have spontaneously differentiated to plasma cells during normal passage in culture (Fig. 6C). Finally, analysis of the levels of secreted IgM in the tissue culture supernatants at 48 hours post-treatment or transfection demonstrated higher levels of secreted IgM in the M2 expressing and LPS treated cultures than in either the M2.Stop (vector) or untreated cultures (Fig. 6D). Thus, we conclude that M2 alone is able to drive plasma cell differentiation of the BCL-1 cell line.

## Discussion

We have shown here that MHV68 reactivation from splenic B cells is linked to M2-driven terminal differentiation of B cells to plasma cells in vivo, supporting previous data that terminal differentiation into plasma cells is linked to reactivation of the human gammaherpesviruses KSHV and EBV [8],[9],[10],[11],[12]. Our previous studies have demonstrated that M2 expression in primary murine B cells in tissue culture was able to drive B cell differentiation along a path toward plasma cells, although during their limited time survival in culture these cells only reached a phenotype referred to as pre-plasma memory B cells [30]. Previous studies on the function of M2 suggest a possible mechanism(s) to drive plasma cell differentiation (Fig. 6E). Madureira et al. [31] and Rodrigues et al. [23] demonstrated that three PXXP motifs located in the C-terminal half of M2 play a role in binding Vav1 and Vav2, and that M2 induces phosphorylation of Vav leading to downstream Rac1 stimulation. Additionally, the M2 protein harbors 2 tyrosine residues that are predicted to be potential phosphorylation sites and have been shown to be essential for the formation of a trimolecular complex with Vav1 and the Src family kinase Fyn [31]. Notably, it has been shown that Vav knockout mice have very low levels of serum immunoglobulin and a severe defect in the induction of Blimp-1 expression [32]. Thus, it seems likely that under some conditions M2 activation of Vav may lead to Blimp-1 expression and entry into the plasma cell program (Fig. 6E). In addition, we have previously shown M2 expression in primary murine B cells leads to high level IL-10 expression [30]. We have proposed to M2-driven IL-10 expression plays a role in both driving expansion of latently infected B cells, as well as suppressing the host immune response during the establishment of latency [30]. However, it is also possible that M2-driven IL-10 expression plays a direct role in facilitating plasma cell differentiation. The latter is based on studies that have shown a role for IL-10 in plasma cell differentiation using model systems employing human B cells [33],[34],[35],[36]. Finally, as we have shown here, M2 also upregulates the expression of IRF4 [we have observed both M2-driven upregulation of IRF4 transcripts (Fig. 6C) and IRF4 protein (data not shown) in BCL-1 cells]. Induction of IRF4 is of significant interest because it has been shown to play critical roles in both isotype switching during the germinal center reaction, and plasma cell differentiation [2],[37]. We propose that entry into the plasma cell program results in the induction of the MHV68 immediate-early transcriptional activator RTA and subsequent activation of the virus replication cycle (Fig. 6E). The latter steps are based on the observed ability of the plasma cell-associated factor XBP-1s to transactivate the critical viral promoters involved in driving expression of the EBV and KSHV lytic switch genes [8],[9],[10],[11],[12].

Does M2-driven plasma cell differentiation play a role in MHV68 reactivation from latently infected M12 B lymphoma cells (see Fig. 1)? We hypothesized that the role of M2 in facilitating TPA reactivation of the latently infected M12 B cell lines could reflect TPA induction of M2 expression, leading to sufficient levels of M2 protein to drive plasma cell differentiation and virus reactivation. This would be consistent with our observation that ectopic expression of M2 alone is sufficient to drive MHV68 reactivation from either wt or M2.Stop infected M12 B cell lines (see Fig. 1D). We have attempted to assess this following TPA stimulation of wild type MHV68 infected M12 cells and have been unable to document any hallmarks of plasma cell differentiation, with the exception of a modest increase in the levels of secreted IgG (XL and SHS, unpublished data). This could reflect insufficient sensitivity of the assays employed – the increased levels of secreted IgG would be consistent with this interpretation, suggesting that a small percentage of cells in the culture differentiate to plasma cells, secrete IgG and then perhaps rapidly disappear due to virus induced cytopathic effect. Alternatively, these results may point to an independent function of M2 that is also involved in promoting virus reactivation from latently infected B cells under some conditions. With respect to the latter possibility, it is clear that plasma cell differentiation is not the only pathway for gammaherpesvirus reactivation from latently infected B cells – other stimuli such as DNA damage can also trigger MHV68 reactivation [38]. A role for M2 in these responses remains to be determined.

It is notable that loss of M2 leads to alterations in immunoglobulin isotype switching in infected B cells (see Fig. 3E and 3F). These results are consistent with our previous observation that M2 null mutants exhibited a significantly slower decay in naïve B cells (CD19+/IgD+) compared to wild type MHV68 [18], suggesting that M2 is involved in driving the differentiation of naïve B cells. Similarly, Simas and colleagues noted that the absence of M2 led to a prolonged persistence of virus infected B cells in germinal centers [22]. Taken together, these data implicate a role for M2 in facilitating B cell differentiation through the germinal center reaction and perhaps directly impacting immunoglobulin isotype switching. Among the genes that were upregulated upon expression of M2 in the BCL-1 B lymphoma cell line was the cellular transcription factor IRF4 (Fig. 6C, and unpublished data), which has been shown to be required for both isotype switching as well as plasma cell differentiation [2]. As such, it is possible that M2 modulation of the levels of IRF4 may account for the apparently distinct roles of M2 in immunoglobulin isotype switching and plasma cell differentiation. This will require further investigation to identify the relevant cellular pathways that are manipulated by M2.

Previous studies have demonstrated that M2 is completely dispensable for virus replication in permissive cell lines, as well as in the lung following intranasal inoculation [18],[39]. However, we have shown a more rapid clearance of M2 null virus replication in the spleen following high dose intraperitoneal inoculation (equivalent replication of wild type and M2 null viruses at day 4 post-infection, and a >20-fold decrease in virus titer of M2 null mutants compared to wild type MHV68 at day 9 post-infection) [21]. The basis for the acute replication defect in the spleen is unknown, but based on a requirement for B cells to seed splenic latency following intranasal virus inoculation (but not following intraperitoneal inoculation) [13],[40], we have postulated a role for virus reactivation from latently infected B cells playing a role in seeding acute virus replication in the spleen. Thus, while high dose intraperitoneal inoculation bypasses the requirement for B cells to seed initial acute MHV68 replication in the spleen at day 4 post-infection [13], virus reactivation from MHV68 infected plasma cells may play a role in driving the sustained virus replication observed in the spleen at day 9 post-intraperitoneal inoculation (levels of virus in the spleen at days 4 and 9 post-infection are equivalent). If so, then the B cell reactivation defect observed in M2 null virus infected mice would translate as a late stage acute virus replication defect in the spleen. Regardless, there is no evidence that M2 plays a direct role in virus replication. As such, M2 appears to play a specialized role to facilitate virus reactivation from latently infected B cells.

An alternative model that is worthy of consideration relates to the impact of M2-driven IL-10 expression on virus-specific CD8+ T cell responses. We have previously shown that loss of M2 leads to increased levels of virus-specific CD8+ T cells, as assessed using tetramers to two distinct viral epitopes (ORF6_486–498_ and ORF61_524–531_) expressed during MHV68 replication [30]. Thus, enhanced CD8+ T cell responses directed against MHV68 replication-associated antigens in M2.Stop infected mice may result in the rapid clearance of virus infected plasma cells expressing replication-associated viral antigens - leading to the observed absence of infected plasma cells at the peak of viral infection in the spleen. As such, in this model, M2 would not be expected to be playing a direct role in driving plasma cell differentiation. We believe that this model is unlikely to account for the absence of MHV68 infected plasma cells based on the observed role of M2 in virus reactivation from the MHV68 M12 infected cell lines (see Fig. 1B and 1D), as well as the ability of M2 to drive terminal differentiation of the BCL-1 cell line in culture (Fig. 6). However, we clearly cannot dismiss a role for enhanced antiviral CD8+ T cell responses in M2.Stop infected mice in controlling the frequency of virus infected plasma cells.

Based on the analysis of M2 function, we propose that M2 falls into a new class of herpesvirus genes that do not directly impact virus replication, but rather facilitate virus reactivation from latency by manipulating cellular differentiation/activation leading to a reactivation competent cellular environment. We have adopted the term reactivation conditioner for such genes. In the case of viruses that establish latency in memory lymphocytes, it is attractive to speculate that it may be necessary to encode functions that drive quiescent memory B or T cells into a state which is more conducive to virus replication. With respect to latency established in memory B cells, plasma cells would appear particularly well suited to support herpesvirus replication. As such, we hypothesize that manipulation of plasma cell differentiation leading to virus reactivation from latently infected memory B cells is relevant to reactivation of the human gammaherpesviruses. Although there is no obvious M2 homolog in either EBV or KSHV, there are several well documented examples of conserved functions encoded by gammaherpesvirus latency-associate gene products that lack obvious sequence homology [41]. Indeed, our previous observation that M2 expression in primary murine B cells triggers IL-6 and IL-10 expression [30], recapitulates functions modulated by both EBV and KSHV [42],[43], and provides further evidence of pathogenic strategies that are conserved among this family of viruses. Importantly, our studies provide the impetus to identify viral gene products encoded by EBV and/or KSHV that manipulate plasma cell differentiation, which may ultimately provide new targets for the development of antiviral therapies against these chronic infections.

## Materials and Methods

### Viruses, cell culture and reagents

The recombinant viruses, generated as described below, were passaged and titered as previously described [44]. Murine NIH 3T12 fibroblast cells were cultured in Dulbecco's modified Eagle medium supplemented with 10% fetal cal serum, 100 U/ml penicillin, 100 µg/ml streptomycin, and 2 mM L-glutamine. The M12 B lymphoma cell line was generously provided by Dr. David Schatz (Yale University School of Medicine, New Haven, CT), and was cultured in RPMI 1640 medium supplemented with 10% fetal cal serum, 100 U/ml penicillin, 100 µg/ml streptomycin and 50 µM 2-mercapto-ethanol.

### Mice and virus infections

Female C57Bl/6 mice 6 to 8 weeks of age were purchased from the Jackson Laboratory. Mice were sterile housed and treated according to Emory University School of Medicine (Atlanta, GA) guidelines and all animal studies were approved by the Emory University Institutional Animal Care and Use Committee. Following sedation, mice were infected intranasally with 100 pfu of either MHV68-YFP or M2stop-YFP viruses in 20 µL of cMEM. Mice were allowed to recover from anesthesia before being returned to their cages.

### Generation of recombinant viruses and latently infected M12 B lymphoma cell lines

The recombinant hygromycin**–**EGFP-expressing MHV68M2.Stop viruses (M2.Stop-HE) were generated by allelic exchange using a previously described MHV68-BAC containing a hygromycin-EGFP fusion gene under the control of the HCMV immediate-early promoter in the ORF27/29b locus (MHV68-HE BAC) [38] and a targeting vector containing the M2 gene with a translation stop and frame shift incorporated at bp 4,559 of the viral genome (pGS284/M2stop) as previously described [45]. To establish MHV68 latently infected M12 cell lines, M12 cells were infected with WT-HE or M2stop-HE viruses by spinoculation at 1,800 rpm for 90 min and then subjected to hygromycin (400 µg/ml) selection. The individual clonal cell lines were established by limiting dilution cloning, and the presence of episomal copies of the MHV68 genome confirmed by Gardella gel electrophoresis [46].

### Immunoblot analyses and plaque assays

Cells were lysed with RIPA buffer (150 mM NaCl, 20 mM Tris.Cl, 2 mM EDTA, 1% NP-40 supplemented with EDTA-free protease inhibitor) (Roche). The whole cell lysates were resolved by SDS-PAGE gel electrophoresis, transferred to nitrocellulose membranes and immunoblotting with chicken anti-ORF59 or rabbit anti-MHV68 antiserum [38]. Plaque assays were performed in NIH3T12 fibroblast cells as previously described [38]. Briefly, cells and supernatants were collected at various times post-induction with TPA (20 ng/ml) and frozen at −80°C. Samples were then subjected to two cycles of freezing and thawing, and virus titers were quantitated by plaque assay on NIH 3T12 fibroblasts.

### RNA isolation and RT-PCR

Total RNA was isolated from untreated or treated cells using TRIzol per manufacturer's protocol (Invitrogen). 2 µg RNA was used for first-strand cDNA synthesis (Invitrogen), followed by PCR amplification using the appropriate oligonucleotide primers as previously described [38]. The primers used for the detection of plasma cell-associated transcripts in BCL-1 cells were as follows; specific spliced XBP-1(s): 5′-GTAGCAGCGCAGACTGCTCGAGATAG-3′ and 5′-GAGGTGCACATAGTCTGCACCAGC-3′, unspliced XBP-1(u): 5′-GTAGCAGCGCAGACTGC TCGAGATAG-3′ and 5′-AGTGCTGCGGACTCAGCAGACCCGGC-3′, J chain: 5′-ATGAAGACC CACCTGCTTCTC-3′ and 5′-GTCAGGGTAGCAAGAATCGG G-3′, IRF4: 5′-ATGAACTTGGA GACGGGCAGCCGGGGC-3′ and 5′-TCACTCTTGGA TGGAAGAATGACGGAGGGA-3′, Blimp-1: 5′-GGAGGATCTGACCCGAATCA-3′ and 5′-CTCCACCATGGAGGTCACATC, M2: 5′-ATGG GCCCAACACCCCCACAAGGAAAG-3′ and 5′-TTACTCCTCGCCCCACTCCACAAAACC-3′, actin: 5′-TAAGTGGTTACAGGAA G-3′ and 5′-AGCCTTCATACATCAAG-3′. All primers employed were designed to amplify spliced gene products and, as such, any products arising from contaminating DNA would run at a larger size (not detected).

### Limiting dilution assays to determine frequency of cells harboring viral genome and reactivating virus

The frequency of MHV68 genome-positive cells was determined using a previously described nested PCR assay (LD-PCR) [13]. Briefly, cells were counted, resuspended in an isotonic solution, and diluted into a background of 10^4^ uninfected NIH 3T12 cells. Following cell lysis with proteinase K, two rounds of nested PCR were performed on each sample to detect the presence of the MHV68 ORF50. To ensure sufficient sensitivity of the nested PCR reaction, 10, 1, or 0.1 copies of a gene 50 containing plasmid (p*Bam*HI N) were diluted into a background of 10^4^ uninfected cells and analyzed in parallel with the experimental sample. The frequency of MHV68 reactivation from latency was also detected as previously described [13]. Briefly, cells were plated in a series of twofold dilutions onto MEF monolayers in 96-well tissue culture plates. After 21 days, wells were scored microscopically for the presence of viral cytopathic effect (CPE). To detect preformed infectious virus, parallel samples were subjected to mechanical disruption as previously described [13], a process that kills >99% of cells without affecting the preformed MHV68 virions [13]. Disrupted cells were plated in a similar series of twofold dilutions.

### Flow cytometry

Flow cytometry analyses for murine splenic cells were done as previously described [30] with the following antibodies: GL-7-Biotin, Stratavidin-APC, CD95-PE, CD138-PE, B220-Pacific Blue (CALTAG Laboratories), CD19-FITC, CD3e-PerCP, IgG2a-Biotin (BD Pharmingen, except where noted). The data were collected on a LSRII flow cytometer (BD Biosciences). For purification of plasma cells, single-cell suspensions were isolated from infected spleens at day 16 post-infection with 100 pfu of the MHV68-YFP recombinant virus administered via intranasal inoculation. The cells were labeled with CD138-PE and B220-Pacific Blue on ice for 20 min, followed by washing with 1% BSA/PBS. The stained cell populations were then sorted on either a FACSVantage or FACSAria™ II flow cytometer (BD Biosciences). For purification of YFP+ cell populations, the single cell suspensions were directly subjected to separation on a FACSVantage and FACSAria™ II flow cytometer (BD Bioscience).

### Enzyme-linked immunosorbent assays (ELISAs) and enzyme-linked immunosorbent spot (ELISPOT) analyses

ELISA for IgM secretion was performed as per manufacturer's protocol (Bethyl Laboratory). To analyze antibody secretion from sorted YFP+ or plasma cell populations, mouse IgG capture antibodies were coated onto a PVDF-backed microplate. The sorted cell populations were plated onto blocked plates at serial dilution and incubated in a humidified 37°C CO_2_ incubator for overnight. The wells were washed and bound antibodies were detected with anti-mouse IgG antibodies. The spots were counted using automated ELISPOT reader.
